# Comparison of Individual and Pooled Stool Samples for the Assessment of Soil-Transmitted Helminth Infection Intensity and Drug Efficacy

**DOI:** 10.1371/journal.pntd.0002189

**Published:** 2013-05-16

**Authors:** Zeleke Mekonnen, Selima Meka, Mio Ayana, Johannes Bogers, Jozef Vercruysse, Bruno Levecke

**Affiliations:** 1 Department of Medical Laboratory Sciences and Pathology, College of Public Health and Medical Sciences, Jimma University, Jimma, Ethiopia; 2 Department of Virology, Parasitology and Immunology, Faculty of Veterinary Medicine, Ghent University, Merelbeke, Belgium; 3 Applied Molecular Biology Research (AMBIOR) Group, Laboratory of Cell Biology and Histology, Antwerp University, Antwerp, Belgium; London School of Hygiene & Tropical Medicine, United Kingdom

## Abstract

**Background:**

In veterinary parasitology samples are often pooled for a rapid assessment of infection intensity and drug efficacy. Currently, studies evaluating this strategy in large-scale drug administration programs to control human soil-transmitted helminths (STHs; *Ascaris lumbricoides*, *Trichuris trichiura*, and hookworm), are absent. Therefore, we developed and evaluated a pooling strategy to assess intensity of STH infections and drug efficacy.

**Methods/Principal Findings:**

Stool samples from 840 children attending 14 primary schools in Jimma, Ethiopia were pooled (pool sizes of 10, 20, and 60) to evaluate the infection intensity of STHs. In addition, the efficacy of a single dose of mebendazole (500 mg) in terms of fecal egg count reduction (FECR; synonym of egg reduction rate) was evaluated in 600 children from two of these schools. Individual and pooled samples were examined with the McMaster egg counting method. For each of the three STHs, we found a significant positive correlation between mean fecal egg counts (FECs) of individual stool samples and FEC of pooled stool samples, ranging from 0.62 to 0.98. Only for *A. lumbricoides* was any significant difference in mean FEC of the individual and pooled samples found. For this STH species, pools of 60 samples resulted in significantly higher FECs. FECR for the different number of samples pooled was comparable in all pool sizes, except for hookworm. For this parasite, pools of 10 and 60 samples provided significantly higher FECR results.

**Conclusion/Significance:**

This study highlights that pooling stool samples holds promise as a strategy for rapidly assessing infection intensity and efficacy of administered drugs in programs to control human STHs. However, further research is required to determine when and how pooling of stool samples can be cost-effectively applied along a control program, and to verify whether this approach is also applicable to other NTDs.

## Author Summary

Since the last decade, growing awareness of the control of neglected tropical diseases (NTDs) has resulted in worldwide increased pledges of drug donations. However, health care decision makers have a limited repertoire of strategies for a rapid assessment of infection intensity and for checking of drug resistance development. Therefore, we verified whether examination of pooled stool samples provide estimates of intestinal worm infection intensity and drug efficacy comparable to those obtained by examination of individual stool samples. Overall, the results showed that pooled samples provide comparable levels of infection intensity and drug efficacy. We conclude that pooling stool samples holds promise as a means of rapidly appraising the intensity of intestinal worm infections on a population level and of monitoring the efficacy of donated drugs. However, this study was conducted in an endemic region. Further research is required to determine when and how pooling of stool samples can be cost-effectively applied in a control program that is reducing the transmission of disease, and to verify whether this approach is also applicable to NTDs other than studied in this paper.

## Introduction

The soil-transmitted helminths (STHs) *Ascaris lumbricoides*, *Trichuris trichiura*, and the two hookworm species, *Necator americanus* and *Ancylostoma duodenale*, cause the highest burden among all neglected tropical diseases (NTDs), with school-aged children and pregnant women being at highest risk [1]–[3].

Preventive chemotherapy (PC) programs, in which albendazole (400 mg) or mebendazole (500 mg) administered in a single dose are the principal means of control of STH infections in school-aged children, has recently received increased political and scientific attention [4], [5]. The World Health Organization (WHO) has devised a roadmap to guide implementation of the policies and strategies set out in a global plan to combat NTDs (period 2008–2015), and more than 70 pharmaceutical companies, governments, and global health organizations committed to supporting this roadmap [6] in the London Declaration on NTDs in January 2012 by sustaining or expanding drug donation programs [7].

These pledges of drug donations are now in place. However, two factors that might affect the success of these programs have received little attention. First, the therapeutic efficacy of the two benzimidazoles (albendazole and mebendazole) differs across STH species [8]. Both drugs are highly efficacious against *A. lumbricoides*, but albendazole is more efficacious against hookworm, and both drugs are unsatisfactory when used as single regimen against *T. trichiura* infection, although mebendazole is relatively more efficacious [8], [9]. Moreover, therapeutic efficacy can vary across levels of infection intensity, albendazole showing a high efficacy when the intensity of *T. trichiura* is low and poor efficacy when infection levels are high [10]. Second, we are relying on two drugs with the same mode of action, and hence the emergence of anthelmintic resistance as drug donations expand, as substantiated in veterinary medicine, is likely [11]–[13]. For these reasons, it is important to seek for alternative strategies (i) to ensure appropriate choice of drug and regimen, (ii) to monitor anthelmintic resistance, and (iii) to assess the long-term impact of PC programs.

Traditionally, both the assessment of infection intensity and drug efficacy are based on the examination of individual stool samples. However, this strategy impedes the up-scale of epidemiological surveys that is required to support health care decision makers to further maximize the efficiency of PC at nationwide level. A possible alternative to individual stool examination is the examination of pooled stool samples. Pooling samples (e.g., stool, serum, and urine) of the same individual has been found valuable for diagnosis of various pathogens, including *Giardia* [14], *Chlamydia* [15], *Salmonella* [16], and HIV [17]. Studies validating a pooling strategy for human STHs are lacking. In animal health it has been shown that pooling stool samples allows for a rapid assessment of infection intensity and drug efficacy. Pools of up to 10 animals provided estimates of intensity of helminth infections by means of fecal egg counts (FECs) comparable to those obtained by examination of individual stool samples [18], [19]. However, it has also been suggested that pooling of animal stool samples may not be recommended when infections become more aggregated [19]. The effect of the number of samples pooled has not yet been examined.

The main objective of the study reported in this paper was therefore to develop and to evaluate a sampling strategy based on pooling of stool samples. To this end, we assessed the intensity of STH infections across varying epidemiological settings and the efficacy of a single dose of mebendazole (500 mg) on both individual samples and pooled samples (pool sizes of 10, 20, and 60 individual stool samples). The ultimate aim was to facilitate rapid identification of STH infections in epidemiological studies and drug efficacy assessment.

## Methods

### Ethics Statement

Ethical approval was obtained from Ghent University (2011/374), Belgium, and Jimma University (RPGC/09/2011), Ethiopia. The efficacy trial was registered under Clinical Trials.gov identifier B670201111554. The school authorities, teachers, parents, and the children were informed about the purpose and procedures of the study. The written consent form was prepared in two commonly used local languages (Afaan Oromo and Amharic) and handed over to the children's parents/guardians. Only those children (i) who were willing to participate and (ii) whose parents or guardians signed the written informed consent form were included in the study. Moreover, an additional separate written informed consent form for children older than 12 years was prepared, read, and handed over to them and their additional written informed consent obtained.

### Study Area and Study Population

The study was conducted in Jimma Town, Ethiopia, located approximately 350 km southwest of the capital, Addis Ababa. Jimma Town is situated at a latitude and longitude of 7°40′N36°50′E, and is characterized by a semi-arid type climate with an average annual rainfall of 800–2,500 mm. The mean daily temperature is 19°C, and ranges from 12 to 30°C. It is located 1,720–2,010 m above see level. Our study focused on schoolchildren from age 5 to age 18, across all eight grades. In total, there were 24 primary schools hosting a total of 23,492 children of all age groups of interest. The female/male ratio across the different schools was approximately 1∶1 (Report Document 2011/2012 of Jimma Education Bureau). STH infections have been documented in Jimma Town, but at present no PC program to control STHs in school-aged children has been implemented.

### Study Design

#### Assessing infection intensity

The assessment of infection intensity of STHs was embedded in a larger ongoing epidemiological survey in Jimma Town that aimed to assess (i) STH prevalence in order to determine frequency of PC, and (ii) seasonal differences in STH prevalence and infection intensity (wet *vs.* dry seasons). For this survey, it was estimated based on varying epidemiological scenarios that at least 120 subjects per school (60 per season) were required for a reliable estimate of apparent prevalence and infection intensity at the school level. The present study assessed the intensity of STH infections in the dry season, between February and March 2012.

**Figure 1:**
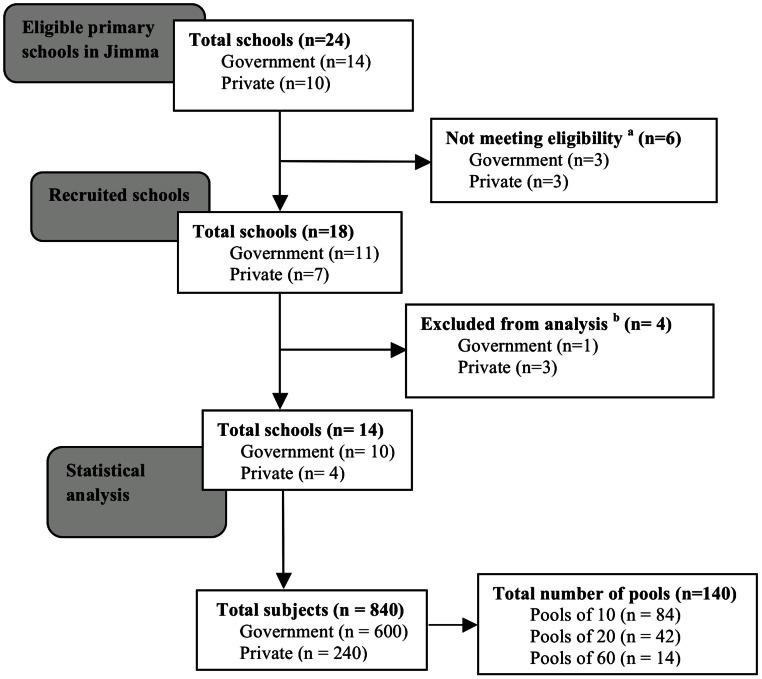
Number of schools and stool samples for assessing infection intensity of STHs in Jimma, Ethiopia. ^a^Eligibility criterion for the schools was hosting grade 1–8 students (age from 5–18 years); ^b^Excluded from analysis since <60 samples were provided.

To this end, all primary schools in Jimma Town hosting all eight grades of students were invited to participate. In each school subjects were stratified according to three age classes (age class A: age 5–9 years, B: age 10–13 years, and C: age 14–18 years). For each age class at least 20 subjects were selected on a voluntary basis, resulting in a total of at least 60 subjects per school. The subjects were asked to provide at least 5 g of stool. This quantity of stool was required to examine the samples individually (2 g) and to pool individual stool samples (1 g). All samples were processed with the McMaster egg counting method (analytic sensitivity of 50 eggs per gram (EPG)) for detection and enumeration of STH eggs [20]. Figure 1 illustrates the number of primary schools eligible, recruited, and included in the statistical analysis.

#### Monitoring drug efficacy

In December 2011, the efficacy of a single dose of mebendazole (500 mg) against STHs was evaluated both on individual and pooled samples. This study was part of a multi-country trial designed to assess the efficacy of a single dose of mebendazole (500 mg; Vermox, Johnson & Johnson, lot no. BCL2F00). To this end, a protocol previously described for assessing drug efficacy of a single dose of albendazole (400 mg) was applied [21]. In short, school children aged 5 to 18 years at different study sites were asked to provide a stool sample during the pre-intervention survey. A single dose of mebendazole was administered to all subjects, regardless of their infection status of STHs. Stool samples were processed using the McMaster egg counting method for the detection and the enumeration of STH infections. Fourteen days post-treatment, stool samples were again collected and processed by the McMaster egg counting method.

**Figure 2:**
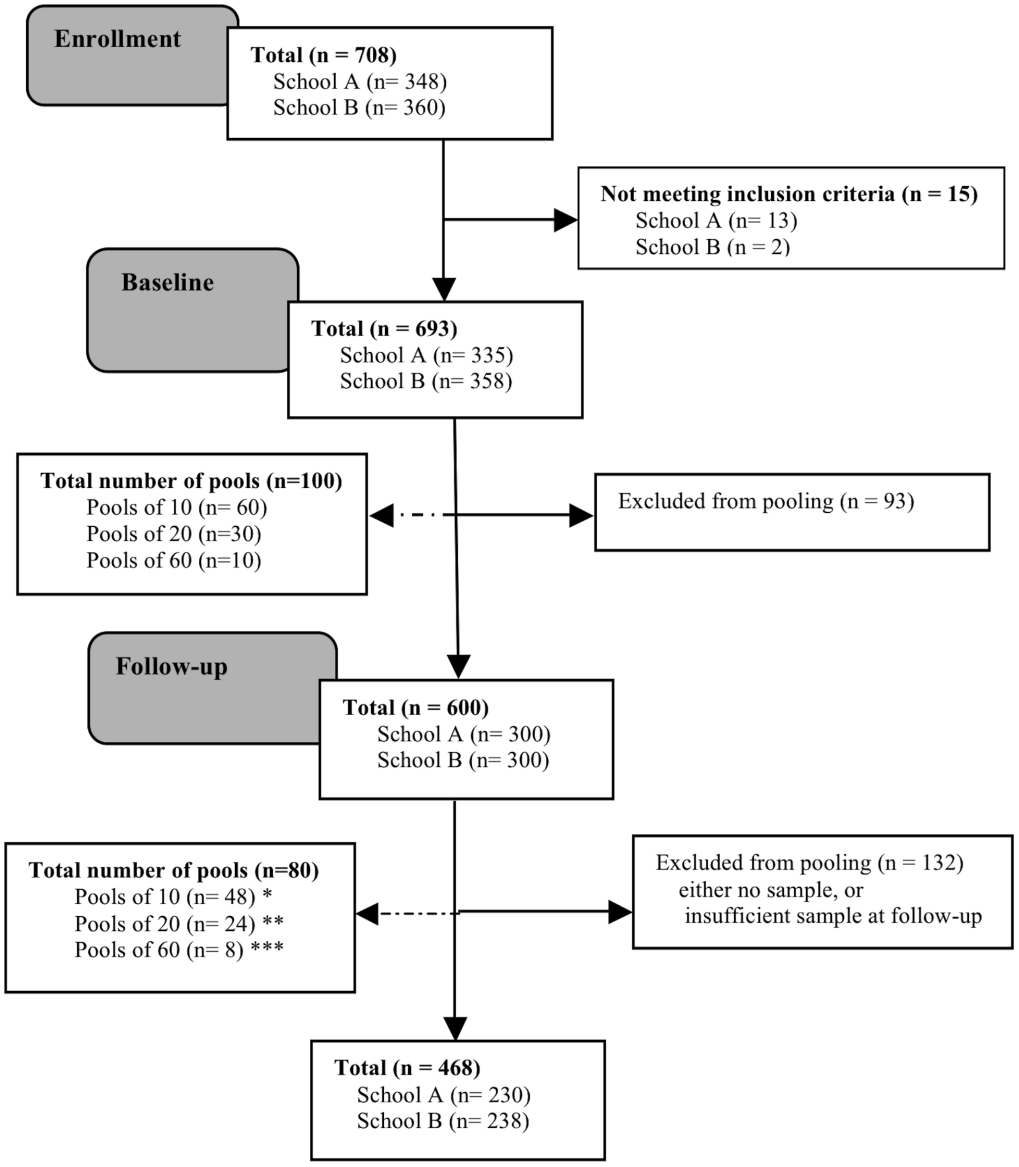
Participation and compliance in assessing drug efficacy against STHs in schoolchildren in Jimma, Ethiopia. At follow-up, pooling of 10, 20, or 60 samples was not always possible: * pool_10_ includes: pools of 9 (n = 12) and pools of 10 (n = 36) samples; ** pool_20_ includes: pools of 18 (n = 5), pools of 19 (n = 2) and pools of 20 (n = 17) samples; and *** pool_60_ includes: pools of 55 (n = 1), 56 (n = 1), 58 (n = 1) and 59 (n = 1) and pools of 60 (n = 4) samples.

This Ethiopian trial was conducted in two schools in Jimma that have previously shown high prevalence rates of STHs [21], [22]. Subjects who were unable to provide a stool sample at baseline, were experiencing a severe concurrent medical condition, had diarrhea at the time of the first sampling, had a known history of allergic reaction to mebendazole, or were pregnant were all excluded from the study. Pregnancy was ruled out based on the following criteria: (i) date of last menstrual period, (ii) sexual intercourse after the last menstrual period, and (iii) correct use of a reliable contraceptive method. Figure 2 summarizes the study subjects enrolled, and followed-up, and the sample submission compliance and number of pooled samples (both at baseline and follow-up) included in the analysis.

### Parasitological Examination

All stool samples were individually processed by the McMaster egg counting method, as described elsewhere [20]. McMaster is a flotation technique that is commonly used in veterinary parasitology both to assess intensity of gastro-intestinal parasite infections and to evaluate drug efficacy against these parasites. For the diagnosis and enumeration of STHs in public health, it has been found to be user-friendly (*vs*. FLOTAC [23]), robust (*vs.* Kato-Katz thick smear) and accurate for enumeration of STHs, but less sensitive when intensity of infection is low (*vs.* Kato-Katz and FLOTAC) [20]. Briefly, 2 g of stool were suspended in 30 ml of saturated salt (NaCl) solution at room temperature (density: 1.2). The fecal suspension was poured three times through a tea sieve to remove large debris. After thorough mixing 10 times, 0.5 ml aliquots were added to each side of a McMaster slide chamber. Both chambers were examined under a light microscope using 100× magnification and the FEC, expressed as EPG for each helminth species, was obtained by multiplying the total number of eggs counted under the microscope by a factor 50. A detailed tutorial can be found on http://www.youtube.com/watch?v=UZ8tzswA3tc.

**Figure 3:**
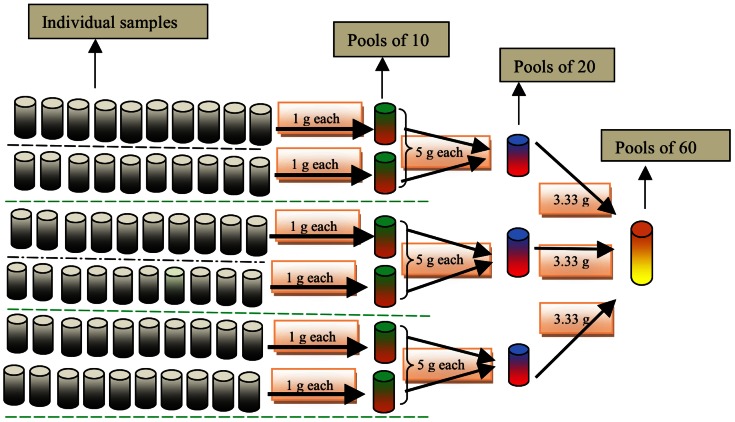
Procedure to obtain pools of 10, 20, and 60 individual stool samples. Sixty individual samples were arranged in 6 rows with each row consisting of 10 individual samples, subsequently 6 pools of 10, 3 pools of 20, and 1 pool of 60 individual samples, resulting in total of 10 pooled samples per school.

In addition, in both studies, a subset of the stool samples was pooled in pools of 10, 20, and 60 individual samples. We considered pooling a rapid alternative to individual stool examination if at least 10 samples were pooled. Pools of 60 individual samples allowed for pooling all stool samples of one school in the study assessing infection intensity. For uniformity across the two studies, pooling of 60 stool samples was also applied for the evaluation of the drug efficacy. Pools of 20 samples were considered as an intermediate between pools of 10 and 60. The procedure for pooling individual samples is illustrated in Figure 3, and is discussed in more detail below. A visualized tutorial can be found on http://www.youtube.com/watch?v=IUZijtBABn0.

At first, 60 individual samples were randomly organized in 6 rows of 10 individual stool samples. From each row 1 g of each of the 10 individual stool samples was transferred into a new pre-labeled plastic beaker (resulting in a total of 6 pools of 10 individual stool samples). After homogenization, 5 g from 2 plastic beakers representing pools of 10 individual samples were transferred into another new pre-labeled plastic beaker, resulting in a total of 3 pools of 20 individual samples. Next, 3.33 g was transferred from the 3 vials of pools 20 into new pre-labeled plastic beaker, resulting in 1 pool of 60 individual stool samples. Finally, each of the pools was processed by the McMaster egg counting method as done for individual samples. Homogenization was standardized by means of stirring the stool until homogenized. Stools from different subjects have different colors. We stopped stirring the pooled stool when the pool had one homogeneous color.

This pooling procedure has two important advantages. First, the cascade procedure applied (e.g. we pooled pools of 10 to make pools of 20) allowed for pooling samples into different pool sizes with only 1 g per individual sample. Second, it avoids the homogenization of too large quantities of stool. For example, for pools of 60 we only had to homogenize 10 g of stools (3.33 g of three pools of 20 individual stool samples), whereas this would have been 60 g if we had pooled 60 times 1 g of individual samples.

For the assessment of infection intensity, samples were randomized according to age class (2 rows of 10 samples per age class). For the efficacy trial, small deviations from the aforementioned procedure should be noted. Although samples were randomly pooled at both baseline and follow-up, unforeseen dropouts meant that pools at baseline did not always match pools at follow-up, when pooling of exactly 10, 20, or 60 samples was not always possible. In addition, not all subjects were included at both baseline and follow-up.

Quality of the parasitological examination was ensured by (i) analyzing the samples within an average of 4 hours, (ii) verification of density of the NaCl solution, (iii) verification of the sensitivity of the scale weighing the fecal material, (iv) supervision of the McMaster and pooling procedures, and (v) re-examination of 10% of the McMaster slides by a senior researcher. The total numbers of the individual samples and pooled samples across the assessment of the infection intensity and the efficacy trial are provided in Figures 1 and 2, respectively.

### Statistical Analysis

#### Assessing infection intensity

The infection intensity was determined for *A. lumbricoides*, *T. trichiura* and hookworm, and expressed as EPG of stool for each individual and each pooled sample. A total of 140 pools (84 pools of 10, 42 pools of 20, and 14 pools of 60) consisting a total of 840 individual samples were pooled. Subsequently, the agreement between mean FEC based on the examination of individual samples and the FEC based on the examination of the pooled sample was evaluated by the Spearman rank correlation coefficient (SAS 9.3 SAS Institute Inc.; Cary, NC, USA). In addition, a permutation test (10,000 iterations) was applied to test for differences in mean FEC between examination of individual and pooled samples. The level of significance was set at *p*<0.05.

#### Monitoring drug efficacy

The efficacy of single dose of mebendazole (500 mg) treatment regimen was evaluated quantitatively based on fecal egg count reduction (FECR; synonym of egg reduction rate), using the following formula:
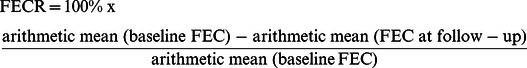



At baseline a total of 600 individual samples were pooled into 60 pools of 10, 30 of 20, and 10 of 60 individual samples, whereas at follow-up 468 individual samples were pooled into 80 pools. As highlighted above, the pool size did not always include the anticipated number of individual stool samples (see also Figure 2). FECR was calculated for the three STHs separately for each of the three pool sizes. A permutation test (10,000 iterations) was applied to test for differences in FECR based on individual and pooled samples. The level of significance was set at *p*<0.05.

## Results

### Prevalence and Infection Intensity

The prevalence of STHs in the 14 primary schools was 52%. *T. trichiura* was the predominant species (39%), followed by *A. lumbricoides* (24%) and hookworm (11%). The arithmetic mean FEC was 2,411 EPG (0–176,000), 295 EPG (0–19,350) and 35 EPG (0–950) for *A. lumbricoides, T. trichiura* and hookworm, respectively. Across schools, there was large variation both in prevalence of any STH infection (11% to 73%) and in prevalence of each of the three STHs (6% to 58% for *T. trichiura;* 0% to 43% for *A. lumbricoides*; and 0% to 30% for hookworm). Across the three age classes there was little variation in prevalence of STHs (age class A: 50% to age class C: 49%), of *T. trichiura* (age class A: 40% to age class C: 36%), and of *A. lumbricoides* (age class A: 28% to age class C: 21%), but substantial variation for hookworm (age class A: 8.0% to age class C: 16%).

### Correlation in Infection Intensity

**Figure 4:**
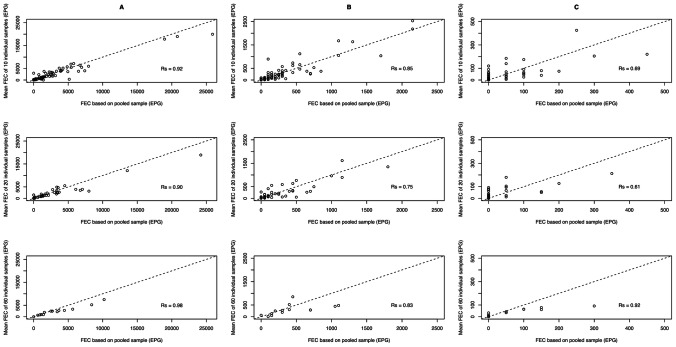
Agreement in FEC of STHs between individual and pooled samples. Each of the 9 scatterplots represents the agreement in mean individual FEC and pooled FEC of stool samples. The plots in column A, B and C represent the *A. lumbricoides*, *T. trichiura*, and hookworm, respectively. The plots in top, middle and bottom row represent pool sizes of 10, 20 and 60, respectively. The magnitude of correlation for each plot is based on the Spearman correlation coefficient (Rs).

Overall, there was a significant positive correlation between mean FEC of individual samples and the FEC of the pooled samples for each of the three STH species (Rs*_A. lumbricoides_* = 0.91, *p*<0.01; Rs*_T. trichiura_ = *0.82, *p*<0.01; Rs_hookworm_ = 0.68, *p*<0.01). As illustrated in Figure 4, there was also a significant positive correlation between mean FEC of individual samples and the FEC of the pooled samples for each of the three pool sizes (*A. lumbricoides*: Rs* = *0.91–0.98, *p*<0.01; *T. trichiura*: 0.75–0.85, *p*<0.01; hookworm: Rs* = *0.62*–*0.92, *p*<0.01).

### Difference in Infection Intensity

Table 1 summarizes the mean FEC for both individual and pooled samples. Overall, there were no significant differences in FEC between individual and pooled samples across the three STHs. Only for *A. lumbricoides* was a significant difference in FEC observed when pool sizes increased up to 60 samples, resulting in higher FECs (FEC_60_ = 3,321 EPG *vs.* FEC_individual = _2,411 EPG, *p*<0.01). For the remaining two STHs, no significant difference across pool sizes was observed (*p*>0.05).

**Table 1:** Mean fecal egg counts for STHs based on individual and pooled samples.

Pool size	Sample size	*A. lumbricoides*		*T. trichiura*		Hookworm	
		Mean FEC (EPG)	*p* value	Mean FEC (EPG)	*p* value	Mean FEC (EPG)	*p* value
**1**	840	2,411	_	295	_	35	_
**10**	84	2,604	0.15	289	0.80	36	0.90
**20**	42	2,842	0.07	312	0.61	27	0.30
**60**	14	3,321	0.01	428	0.09	64	0.08

EPG, eggs per gram of stool; FEC, fecal egg count;

### Monitoring Drug Efficacy

The mean FEC and FECR for each of the STHs based on examination of individual and pooled samples are described in Table 2. Based on individual samples, FECR was high for *A. lumbricoides* (97.2%), but only moderate for *T. trichiura* (60.9%) and low for hookworm (44.2%). Pooled samples provided comparable FECR results for *A. lumbricoides* and *T. trichiura*. However, for hookworm, a significant statistical difference was found for pools of 10 (*p* = 0.03) and 60 individual samples (*p* = 0.02).

**Table 2:** Fecal egg count reduction of mebendazole for STHs assessed by pooled and individual samples.

Pool size	Sample size at baseline/follow-up	*A. lumbricoides*			*T. trichiura*			Hookworm		
		Mean FEC at baseline (EPG)	FECR (%)	*p* value	Mean FEC at baseline (EPG)	FECR (%)	*p* value	Mean FEC at baseline (EPG)	FECR %	*p* value
**1**	600/468	3,091	97.2	_	320	60.9		43	44.2	_
**10**	60/48*	3,354	96.8	0.74	338	53.2	0.39	60	68.8	0.03
**20**	30/24**	4,965	96.8	0.80	607	77.7	0.62	67	65.6	0.26
**60**	10/8***	4,020	96.1	0.24	305	57.7	0.44	50	87.5	0.02

At follow-up, pooling of exactly up to 10, 20, or 60 samples was not always possible:

* pool_10_ includes: pools of 9 (n = 12) and pools of 10 (n = 36) samples;

** pool_20_ includes: pools of 18 (n = 5), pools of 19 (n = 2) and pools of 20 (n = 17) samples; and*** pool_60_ includes: pools of 55 (n = 1), 56 (n = 1), 58 (n = 1) and 59 (n = 1) and pools of 60 (n = 4) samples.

EPG, eggs per gram of stool; FEC, fecal egg count; FECR, fecal egg count reduction.

## Discussion

Given the recent pledges of continuing donations of anthelmintic drugs [7], and hence prospects of increasing drug pressure on parasite populations, cost-effective tools to guide healthcare decision makers on how to optimize treatment strategies and on how to monitor the control of STHs are urgently needed. In analogy with studies conducted in animal health, our results show that pooling stool samples also holds promise as a rapid strategy in public health (i) to assess infection intensity, (ii) to ensure appropriate choice of drug and regimen, (iii) to monitor anthelmintic resistance, and (iv) to assess the long-term impact of the ongoing PC programs to control STHs.

However, before we can provide specific recommendations, further research is required to gain additional insights into how and when to apply pooling as described here. First, it is essential to assess the effect of pool size, sample size (number of pools), the detection limit of the diagnostic method for quantifying infection intensity by means of FEC (FEC method), and level of aggregation and intensity of infections on the precision and the accuracy of FEC and FECR results. This last assessment is particularly essential when level of infection and aggregation of STH infections change across different rounds of PC, and hence demand a different pool and sample size and FEC method. This effect of level of infection and aggregation of STH infections on factors inherent to the study design (pool size, sample size and detection limit of the FEC method) became already apparent in the present study, where pooling samples to assess drug efficacy using the McMaster egg counting method worked for *A. lumbricoides* and *T. trichiura*, but not for hookworm, for which the level of FEC was low and FECs were highly aggregated. Because it is impossible to thoroughly evaluate the impact of pool size, sample size, the detection limit of the FEC method, and level of aggregation and intensity of infections by field or laboratory experiments, a simulation study is in place. This approach will allow us to theoretically assess the accuracy and precision of FEC and FECR across different scenarios of pool size, sample size, detection limit of the FEC method, and level of aggregation and intensity of infections. Recently, such a simulation study has been performed for FECR based on individual stool samples [19], [24], which can be easily adapted for a pooling strategy.

Second, a detailed cost-effectiveness analysis is highly recommended [25]. Examination strategies resulting in a comparable level of accuracy and precision on FEC or FECR may still require a different level of technical and financial support. The present study was not designed to verify the cost-effectiveness of our pooling strategy. However, from the current results obtained in a region endemic to STHs, pooling of stool samples might well be cost-effective. For example, in the present study we were able to reduce the samples examined by a factor of 10 without a significant loss in accuracy of the FEC results (only for *A. lumbricoides* was a significant difference in FEC observed between individual samples and pools of 60). We estimate that processing and reading a McMaster requires approximately 5 min [23]. By pooling 10 individual samples we would save 270 min per day ( = 60 individual samples ×5 min – 6 pools of 10×5 min). Of course, pooling samples requires some additional time, and pooling will be hard to justify as cost-effective when the pooling procedure requires more than 270 min. In the strategy used for the study described here we had to weigh a certain quantity of stool 60 times (60 individual samples to make 6 pools of 10) and to homogenize 6 pools. If we conservatively assume that homogenization of 1 pool demands 5 min, the pooling strategy will still be cost-effective when the quantity of stools can be measured within 240 min (270 – 6 pools of 10×5 min) or 4 min per step of measuring stool (240/60). Given that McMaster can be applied in 5 min and comprises weighing of 2 g of stool, homogenization in a flotation solution, and filling and reading of the McMaster slide, it is clear that the 4 min available to transfer a fixed quantity is also quite conservative.

Third, various strategies for pooling stool samples should be evaluated. In the present study we pooled samples in a cascade: pools of 10 were made by pooling individual samples, but rather than repeating this procedure of pooling individual samples for the other pool sizes, we used the pools of 10 to make pools of 20, and subsequently the pools of 20 to make the pools of 60. This procedure provided an equal amount of stool pooled for each pool, *in casu* 10 g, and an elegant way to assess different pool sizes without too much additional work. However, it remains to be established that this procedure does not itself introduce any bias, particularly when the contribution of each subject decreases over pool size. For pools of 10, each individual in our study contributed 1 g, whereas this was 0.5 g and 0.15 g for pools of 20 and 60, respectively. Moreover, pools were homogenized by simple stirring. Homogenization in a liquid phase prior to examination, however, should be recommended, as this facilitates homogenization of pools. It is particularly important when eggs are not equally distributed among stool samples [26]. Homogenization of stool remains a crucial step in the most important FEC methods applied in veterinary parasitology, including McMaster, FECPAK (www.fecpak.com) and (mini-)FLOTAC [27; unpublished data]. An additional point is that homogenization will reduce drying of the stool while pooling samples. Due to our cascade procedure larger pool sizes were made at the end, but this probably resulted in an increase of evaporation of water from the stool samples. As a consequence of this increase, the mass of the stool decreased over time, whereas the number of eggs in the stool remained unchanged, hence the observed trend of increasing FECs over pool sizes. This potential bias in FECs could have been overcome by homogenizing the pools immediately in the flotation solution. Finally, to further simplify procedures under field conditions, it would be worth evaluating pooling based on a fixed volume rather than pooling a fixed amount of feces.

Fourth, notwithstanding the comparable FECR results between individual and pooled stool samples for *A. lumbricoides* and *T. trichiura* in our study, assessing drug efficacy based on pooled samples remains a delicate matter. Subjects who are not infected (truly or apparently) at baseline cannot be excluded from the analysis and there is no perfect match of pools before and after drug administration due to drop out. Therefore, both analyses need to be validated independently.

Fifth, we focused only on STH infections, but since the advocacy to integrate NTD control measures, this pooling strategy should also be validated for other NTDs, such as *schistosomiasis*. Finally, although pooling samples does not provide prevalence data, various models have been developed for other pathogens to estimate prevalence based on pooled samples. Validation of these models for STHs is required [28].

In conclusion, this study highlights that pooling stool samples is a rapid procedure that holds promise as a cost-effective strategy for assessing the intensity of STH infection and for monitoring PC programs. However, further research is required (i) to gain additional insights into the impact of pool size, sample size, detection limit of the FEC method, intensity, and aggregation of infections on the validity of pooling stool samples, (ii) to verify the cost-effectiveness of pooling, (iii) to optimize the methodology of pooling stool samples, and (iv) to validate models to estimate prevalence based on pooled samples.

## Supporting Information

Supporting information S1 **CONSORT checklist**
(DOC)

Supporting information S2 **Trial protocol**
(DOCX)
