# Supplementary material for: Comparison of Individual and Pooled Stool Samples for the Assessment of Soil-Transmitted Helminth Infection Intensity and Drug Efficacy
Source: PLoS Negl Trop Dis. 2013 May 16;7(5):e2189. doi: 10.1371/journal.pntd.0002189 (PMC3656117; doi:10.1371/journal.pntd.0002189)
Supplement: Supporting information S2 — Trial protocol (DOCX) [file pntd.0002189.s002.docx]

**Protocol - Monitoring efficacy of mebendazole for the treatment of Soil Transmitted Helminths**

Table of Contents

1. Background 1

2. Objectives 2

3. Materials and Methods 2

3.1. Study sites and population 2

3.2. Study design 2

3.3. Parasitological techniques 3

3.4. Statistical analysis 3

4. Ethical issues 3

5. Starting date 3

6. References 3

# 1. Background

*Soil-Transmitted Helminths*

The three major Soil-Transmitted Helminths (STH), *Ascaris lumbricoides* (roundworm)*, Trichuris trichiura* (whipworm) and *Necator americanus/Ancylostoma duodenale* (hookworms) are amongst the most widespread parasites worldwide. An estimated 4.5 billion individuals are at risk of STH infection and more than one billion individuals are thought to be infected, of whom 450 million suffer morbidity from their infection, the majority of who are children. An additional 44 million infected pregnant women suffer significant morbidity and mortality due to hookworm-associated anemia. Approximately 135,000 deaths occur per year, mainly due to infections with hookworms or *A. lumbricoides* (Bundy *et al*., 1992; Stoltzfus *et al*., 1996; Crompton *et al*., 2002; WHO, 2005; Bethony *et al*., 2006)*.*

*Treatment and monitoring efficacy*

The periodic administration of anthelmintics is currently the most widely used method to control these infections. Rather than aiming to achieve eradication, these control programs are focused on reducing infection intensity and transmission potential, primarily to reduce morbidity. The benzimidazole (BZ) drugs, i.e. albendazole (ALB) and mebendazole (MBZ), are the most widely used drugs and a scale-up of these large-scale treatment programs underway in various parts of across Africa, Asia and Latin-America (donation of 400 million tablets of albendazole by GlaxonSmithKline and 200 million tablets of mebendazole by Johnson & Johnson). Due to the scarcity of available anthelmintics, it is imperative that monitoring systems are designed both to assess progress and to detect any changes in therapeutic efficacy that may arise from the selection of worms carrying genes responsible for drug resistance. Although some small-scale studies (De Clercq *et al*., 1997; Albonico *et al*., 2004) have suggested a reduced efficacy of these components, these studies should be interpreted with some caution. Published studies are confounded by methodological variations including treatment regimens, poor quality of drugs, differing statistical analyses used to calculate therapeutic efficacy, as well as a range of other problems in study design, such as small sample size, diagnostic methods, variation in pre-intervention infection intensities and confounding factors related to geographical locations. A recent multinational trial standardized across different endemic regions using a protocol which was standardized in terms of the treatment (a single-oral 400mg dose of ALB originating from the same batch), the follow up (between 14 and 30 days after) and the detection technique (the McMaster egg counting technique), indicated that ALB is highly efficacious against *A. lumbricoides* and hookworms, but not for *T. trichiura* (Vercruysse *et al*., 2011). MBZ is likely to be more efficacious against *T. trichiura*, yet reliable efficacy data for this BZ are not available.

# 2. Objectives

The overall objective is to monitor efficacy of MBZ against STH.

The primary objective is:

(1) to monitor the efficacy a single dose 500 mg of MBZ against STH infections by means of Faecal Egg Count Reduction (FECR) and Cure Rate (CR).

# 3. Materials and Methods

## 3.1. Study sites and population

**Study sites**

Six out of the seven study sites involved in the 2009 Study (ALB) confirmed their participation, including Brazil (Minas Gerais State), Cambodia (Phnom Phem), Cameroon (Yaoundé), Ethiopia (Jimma), Tanzania (Pemba Island) and Vietnam (Hanoi). India will be replaced by Argentina (Orean region). Each of these study sites has a documented history of STH and drug selection pressure. Moreover, each of them has well equipped diagnostic facilities, skilled personnel.

**Study Population**

Schoolchildren between 4 and 18 years old are the focus of this study because of two main reasons: School children are normally a major target for regular treatment with anthelminthic, because they are the group that usually has the heaviest worm burdens for *A. lumbricoides* and *T. trichiura*, and are steadily acquiring hookworm infections. In addition, they are in a period of intense physical and intellectual growth (Bundy *et al.,* 1992; Crompton and Nesheim, 2002). Deworming schoolchildren has a considerable benefit on their nutritional status (Stoltzfus *et al*., 1996, Curtale *et al.,* 1995), physical fitness, appetite, growth (Stephenson *et al*., 1993) and intellectual development (Nokes *et al* 1994).

## Study design

**Primary objective**

Following obtaining informed consent, schoolchildren in the target age range group will be recruited and asked to provide a recent stool sample (an interval of less than 4 hours) that will be processed to determine the FEC for each STH present. For the initial sampling the aim is to enroll at least 250 infected children for at least one of the STH. This sample size was selected based on statistical analysis of study power, using random simulations of correlated over-dispersed FEC data reflecting the variance-covariance structure in a selection of real FEC data sets. This analysis suggested that a sample size of up to 200 individuals (α = 0.05, power = 80%) was required to detect a 10 percentage point drop from a null efficacy of ~ 80% (mean percentage FEC ∆ per individual) over a wide range of infection scenarios. Standard power analyses for proportions also indicated that the detection of a ~10 percentage point drop from a null cure rate required sample sizes up to 200 (the largest samples being required to detect departures from null efficacies of around 50%). Given an anticipated non-compliance rate of 25%, a sample of 250 infected subjects was therefore considered necessary at each study site.

All children providing stool samples will be treated with MBZ single table of 500mg under supervision (chewing + water). The MBZ will be provided (free) by the coordinating group. **Seven up to fourteen days (maximum interval)** after treatment a second faecal sample will be collected from the children to determine again FEC. Subjects who are unable to provide a stool sample at follow-up, or who are experiencing a severe concurrent medical condition or have diarrhea at time of the first sampling, will be excluded from the study.

## Parasitological techniques

#### Determination of FEC of STH

All fecal samples were processed using the McMaster egg counting technique (analytic sensitivity of 50 EPG) for the detection and the enumeration of infections with *A. lumbricoides*, *T. trichiura* and hookworms. All study sites are familiar with the technique and McMaster slides were provided previously.

## 3.4. Statistical analysis

Both CR and FECRT will be considered to monitor to efficacy of MB against STH. The statistical analysis will be assessed as described by Vercruysse *et al*., 2011.

# 4. Ethical issues

The overall protocol of the study will be reviewed by the Ethics committee of the Faculty of Medicine, Ghent University, Belgium. For each proposed study a separate ethical clearance need to be obtained.

# 5. Starting date

It is expected to start in end 2011 (if later, please justify)

# 6. References

- Albonico M, Engels D and Savioli L (2004) Monitoring drug efficacy and early detection of drug resistance in human soil-transmitted nematodes: a pressing public health agenda for helminth control. Int J Parasitol 34, 1205-1210.
- Bethony J, Brooker S, Albonico M, Geiger SM, Loukas A, et al. (2006) Soil-transmitted helminth infections: ascariasis, trichuriasis, and hookworm. Lancet 367, 1521-1532.
- Bundy DA, Hall A, Medley GF and Savioli L (1992). Evaluating measures to control intestinal parasitic infections. World Health Stat Q45, 168-179.

Crompton DWT and Nesheim MC (2002) Nutritional impact of intestinal helminthasis during the human life cycle. Annu Rev Nutr 22, 35-59.

- Curtale F, Pokhrel RP, Tilden RL and Higashi G (1995) Intestinal helminths and xerophthalmia in Nepal. A case-control study. J Trop Pediatr 41, 334-337.
- De Clercq D, Sacko M, Behnke JM, Gilbert F, Dorny P, et al. (1997) Failure of mebendazole in treatment of human hookworm infections in the Southern Region of Mali. Am J Trop Med Hyg 57, 25- 30.
- Nokes C and Bundy DA (1994) Does helminth infection affect mental processing and educational achievement? Parasitology Today 10, 14-18.
- Stephenson LS, Latham MC, Adams EJ, Kinoti SN and Pertet A (1993) Physical fitness, growth and appetite of Kenyan school boys with hookworm, *Trichuris trichiura* and *Ascaris lumbricoides* infections are improved four months after a single dose of albendazole. J Nutr 123, 1036-1046.
- Stoltzfus RJ, Albonico M, Chwaya HM, Savioli L, Tielsch J, et al. (1996) Hemoquant determination of hookworm-related blood loss and its role in iron deficiency in African children. Am J Trop Med Hyg 55,399-404.
- Vercruysse J, Behnke JM,Albonico M, Ame SM, Angebault C, et al. (2011) Assessment of the anthelmintic efficacy of albendazole in school children in seven countries where soil-transmitted helminths are endemic. PloS Negl Trop Dis 5: e948.
- World Health Organization (2005) Deworming for health and development. Report of the third global meeting of the partners for parasitic control. WHO/CDS/CPE/PVC/2005.14, World Health Organization, Geneva.
